# Supplementary material for: Phenotyping of Rare CFTR Mutations Reveals Distinct Trafficking and Functional Defects
Source: Cells. 2020 Mar 19;9(3):754. doi: 10.3390/cells9030754 (PMC7140603; doi:10.3390/cells9030754)
Supplement: Supplementary file 1 [file cells-09-00754-s001.pdf]

Supplementary figures

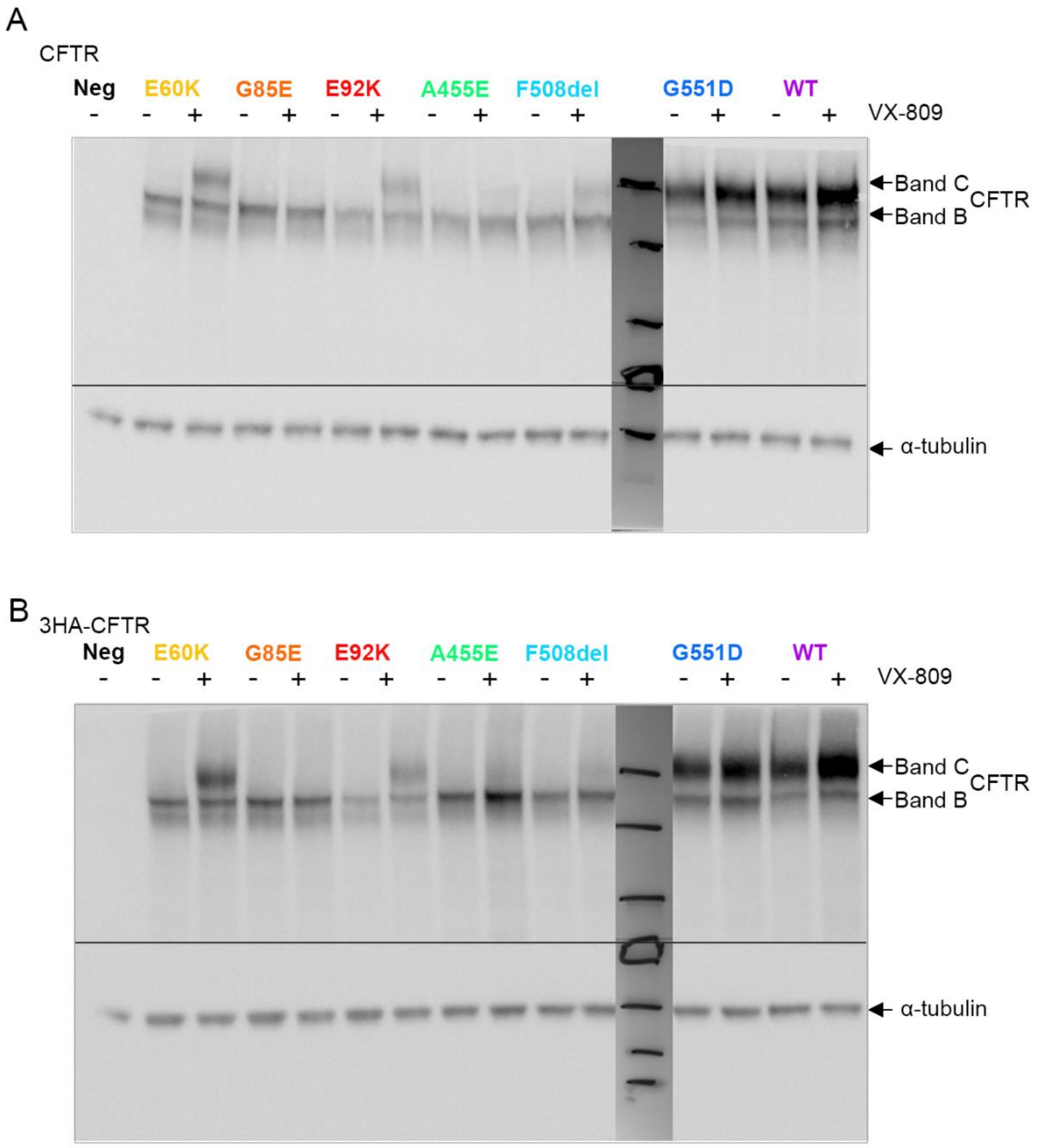

Suppl. figure S1. Assessment of CFTR maturation of rare CFTR mutants.

Uncropped gels of the western blot analysis of (**A**) untagged and (**B**) 3HA-tagged CFTR variants by anti-CFTR antibodies and α-tubulin (loading control) in whole cell lysates of stably transduced HEK293T as shown in figure 1. The lower band (ER, 'band B') represents immature, core-glycosylated CFTR, the upper band (Golgi, 'band C') the complex glycosylated, fully mature protein. The grey line shows were the blot was cut for incubation with different antibodies.

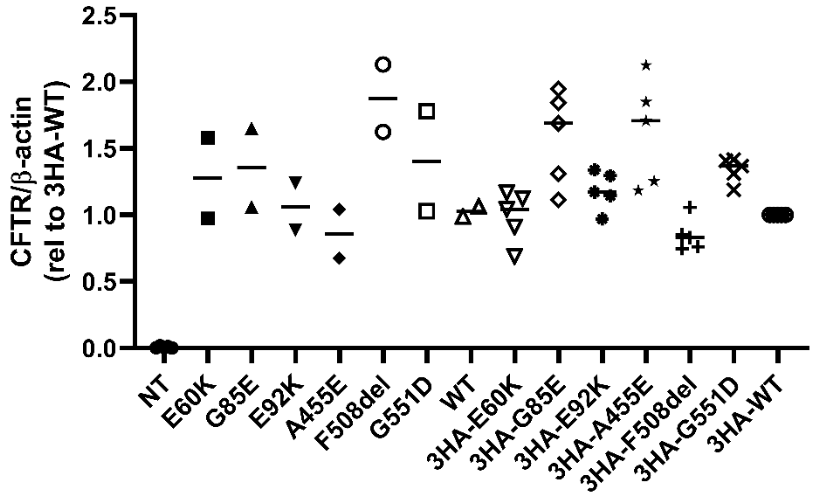

Suppl. figure S2. Quantification of *CFTR* mRNA levels in HEK293T stable cell lines.

For all HEK293T cell lines stably overexpressing untagged or 3HA-tagged CFTR, *CFTR* mRNA levels were determined by RT-PCR using primers specific for the 3′ region of the *CFTR* cDNA. *CFTR* mRNA expression was normalized to β-actin mRNA, and set relative to 3HA-WT. Single data points of independent replicate experiments are depicted, and their mean.

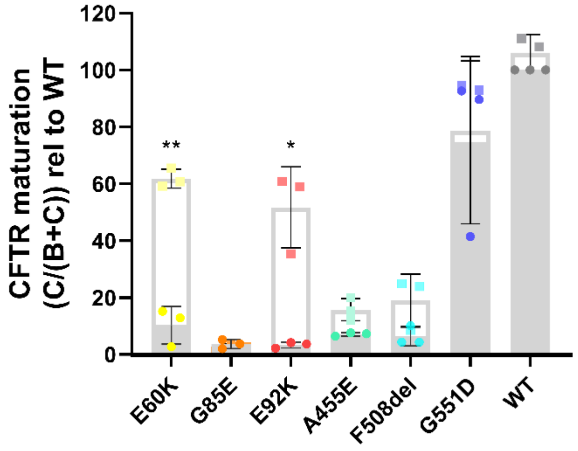

Suppl. figure S3. Quantification of CFTR maturation of 3HA-tagged CFTR variants.

Quantification of figure 1C, showing the rescue in CFTR maturation by corrector VX-809 (2.5 µM, 24h) expressed as the increase in band C over total (B+C) CFTR relative to WT. Graph depicts the mean ± SD of three independent experiments. * $p<0.05$, ** $p<0.01$

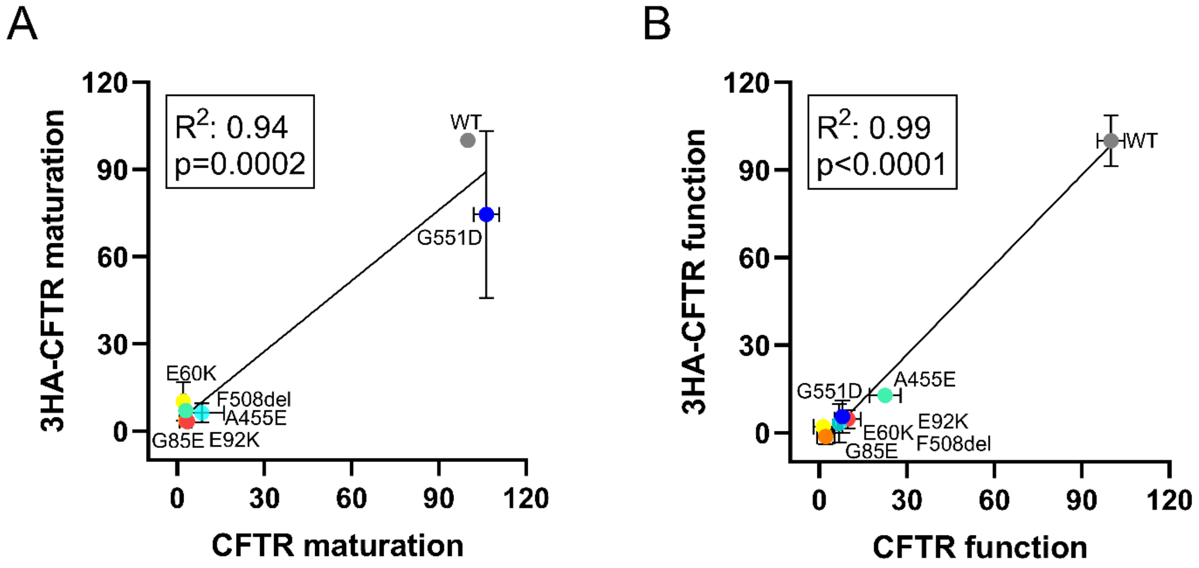

Suppl. figure S4. Pearson correlation of CFTR maturation and function of untagged and 3HA-tagged CFTR variants at baseline.

(**A**) HEK293T cells stably overexpressing untagged or 3HA-tagged CFTR variants were analyzed by western blotting (blots depicted in Fig. 1B, C) and the amount of band C over total (B+C) CFTR relative to WT quantified for both conditions under DMSO treatment. The Pearson correlation of untagged vs. 3HA-tagged CFTR maturation at baseline is depicted. (**B**) Pearson correlation of CFTR function at baseline measured by HS-YFP quenching after stimulation with 10 µM forskolin (Fsk). Graphs depict the mean ± S.D. of three independent experiments.

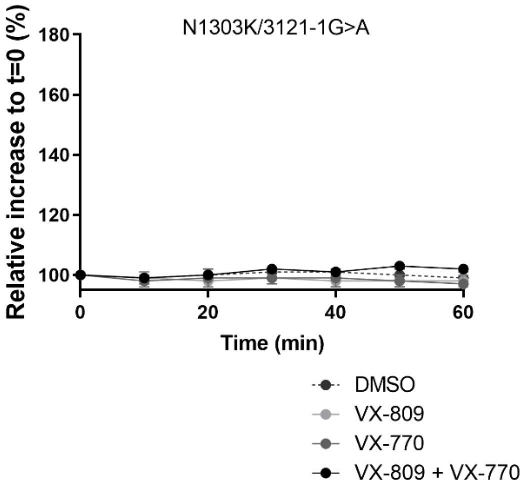

Suppl. figure S5. Assessment of CFTR function in CF intestinal organoids.

FIS responses of CF organoids (genotype N1303K/3121-1G>A) treated with VX-809 (24 h, 2.5 µM), VX-770 (3 µM), VX-809+VX-770 or DMSO, all stimulated with Fsk 5 µM. Time course of 60 min. A representative experiment is shown (n=2 independent repeats). Mean ± S.D. of 4 wells per condition (~300 organoids/well). Abbreviations: FIS: forskolin induced swelling (FIS).

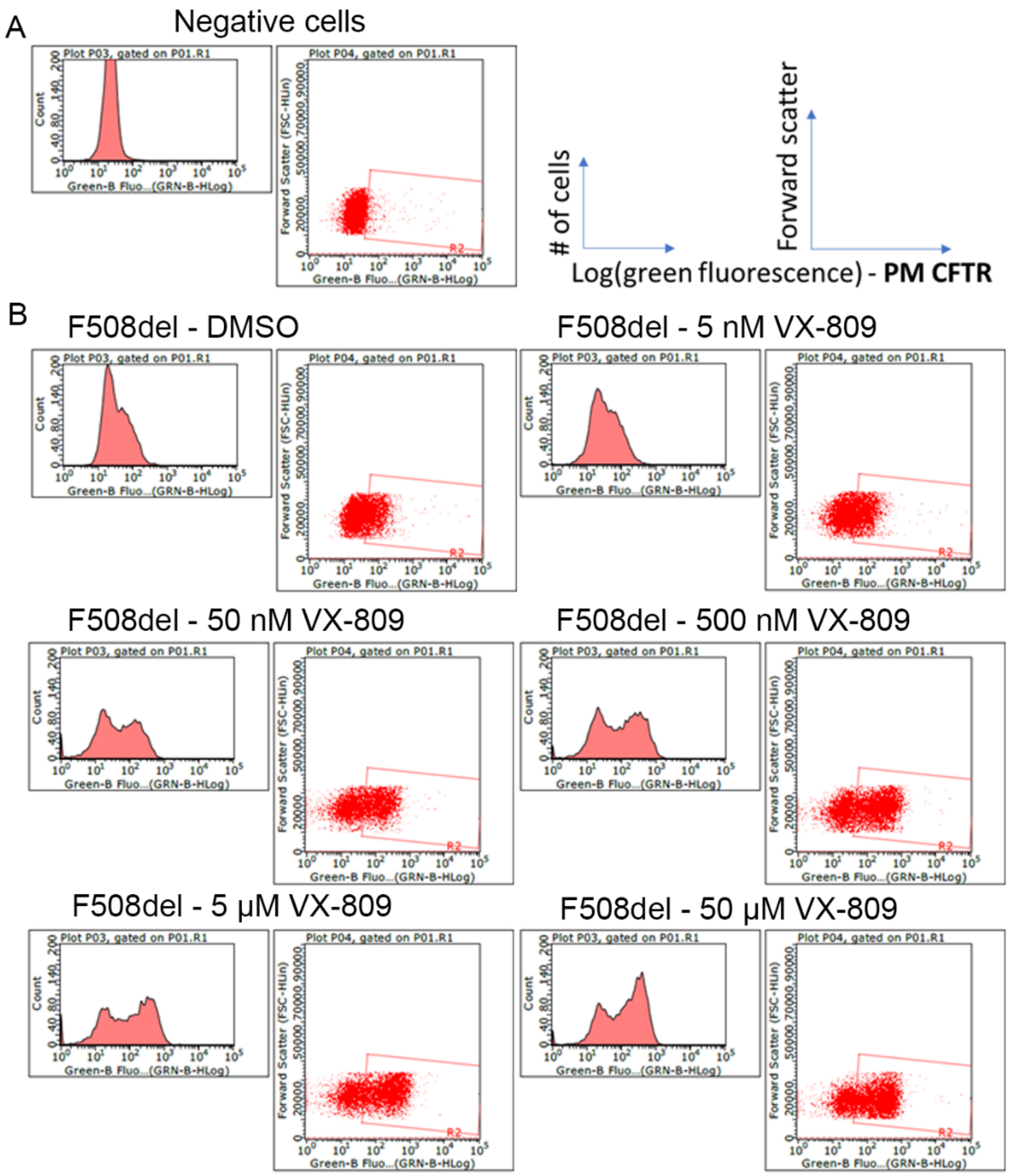

Suppl. figure S6. Plasma membrane density of F508del-CFTR upon treatment with increasing concentrations of corrector VX-809.

Histograms and dot plots (Forward Scatter vs. Log(Green fluorescence)) for plasma membrane (PM) CFTR in (**A**) negative cells (used to position the gate for CFTR positive cells) and (**B**) 3HA-F508del cells treated with increasing concentrations of CFTR corrector VX-809, as also shown in figure 5A. Per condition results from a single, representative well are shown. PM CFTR was detected based on anti-HA detection of 3HA-CFTR (as depicted in Figure 4A).
